# Cattle H5N1 outbreak in US driven by cow’s “cross-nursing” behaviour: elucidating a novel transmission mechanism

**DOI:** 10.1080/22221751.2025.2547736

**Published:** 2025-08-13

**Authors:** Shu Hu, Jie Cui

**Affiliations:** a Shanghai Sci-Tech Inno Center for Infection & Immunity, National Medical Center for Infectious Diseases, Huashan Hospital, Institute of Infection and Health, Fudan University, Shanghai, People’s Republic of China; b Laboratory for Marine Biology and Biotechnology, Qingdao Marine Science and Technology Center, Qingdao, People’s Republic of China

**Keywords:** H5N1, cow, outbreak, cross-nursing, transmission

## Introduction

The emergence of highly pathogenic avian influenza A(H5N1) in US dairy cattle represents an unprecedented zoonotic event with critical pandemic implications. As of June 2025, over 1,070 dairy farms across 17 states have been affected, with documented spillover to humans resulting in 70 confirmed cases. The mechanistic basis for mammary tropism and high viral loads in milk remains unexplained, hampering control efforts.

**Figure 1.**
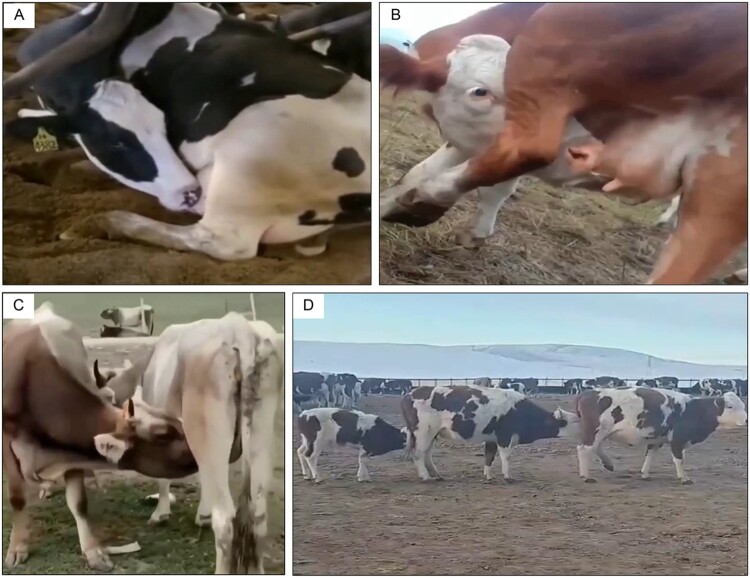
Nursing behaviour of dairy cattle pontentially facilitating viral transmission including auto-nursing (A and B), mutual-nursing (C), and chain-nursing (D). Images were taken from Shi et al. [1], with permission from the leading author.

A recent study led by Prof. Hualan Chen in Harbin Veterinary Research Institute, China elucidates the pathogenesis of H5N1 mammary gland infection through experimental cattle studies [1]. Prof. Chen’s team demonstrate that mammary invasion occurs via “mouth-to-teat” transmission during nursing rather than systemic dissemination following respiratory infection (Figure 1). Crucially, both inactivated and DNA vaccines provided sterilizing immunity against multiple H5N1 strains. These findings define the transmission pathway underlying this zoonotic outbreak and establish vaccination as a viable intervention strategy.

## Novel transmission mechanism

The study demonstrates that H5N1 viral replication in calves’ oral cavities facilitates direct transmission to mammary glands during sucking behaviour, contradicting conventional respiratory transmission models. Systematic experimental approaches revealed that oral viral shedding enables mammary invasion through direct mucosal contact during cross-nursing behaviours observed in 1%−50% of dairy operations affecting 0.5%−40% of individual cattle [2,3].

Comparative exposure studies established oral replication as the critical determinant for mammary transmission, with viral detection in milk and mammary tissues exclusively from animals exposed to orally infected calves. This mechanism explains the unique epidemiological characteristics distinguishing this outbreak from conventional respiratory influenza patterns.

Cattle oral tissues, mammary glands, and teats express both avian-type (α2,3-linked) and human-type (α2,6-linked) sialic acid receptors. This dual receptor expression facilitates H5N1 viral infection through contaminated feed and water while carrying profound implications for viral evolution and pandemic potential [4]. The receptor configuration mirrors that observed in ferret models, potentially facilitating acquisition of human-type receptor binding capabilities that enhance mammalian transmissibility.

## Viral evolution and vaccine efficacy

Molecular characterization of recovered viral isolates revealed amino acid substitutions including PB2-M631L and PB2-E627 K, indicative of selective evolutionary pressure favouring mammalian adaptation. These polymerase gene mutations, previously characterized as enhancing viral replication efficiency in mammalian cellular environments, constitute molecular determinants of host range expansion and elevated zoonotic transmission potential [5,6]. This finding corroborates established paradigms of polymerase-mediated host adaptation documented in H5N1 and H7N9 lineages [7,8].

Strain-specific replication kinetics demonstrated marked heterogeneity, with the broad-tropism TS/23 exhibiting broad tissue tropism and robust replicative fitness, while the restricted-replication DK/24 displayed restricted replicative capacity. This phenotypic divergence underscores the necessity for comprehensive viral surveillance frameworks to monitor evolutionary trajectories [9].

Both inactivated and DNA vaccines achieved sterilizing immunity against heterologous H5N1 challenges, with vaccinated cattle demonstrating complete protection against high-dose respiratory and mammary challenges. Cross-protective efficacy encompassed both Eurasian strains and the cattle-adapted DC/24 isolate, indicating robust coverage within the 2.3.4.4b phylogenetic clade. These findings validate established vaccination paradigms previously employed for containment of H5N1 and H7N9 epizootics in avian populations [10].

Beyond polymerase adaptations, HA and NA substitutions in bovine H5N1 isolates reveal concerning evolutionary trends. Key substitutions like HA-Q226L and HA-G228S [11], which enhance human-type receptor binding, warrant attention given dual receptor expression in cattle oral and mammary tissues. NA stalk deletions in some isolates may also affect viral fitness. Combined with the cross-nursing replication environment, these changes underscore the need for comprehensive genomic surveillance to monitor pandemic risk evolution.

## Conclusions

This investigation resolves the mechanistic puzzle of H5N1 mammary gland invasion while providing evidence-based solutions for disease control. The elucidation of mouth-to-teat transmission through cross-nursing behaviour explains unique epidemiological characteristics and identifies specific intervention targets. The demonstration of sterilizing immunity through vaccination, combined with behavioural management strategies, establishes a comprehensive framework for effective H5N1 control in dairy operations, providing practical solutions for this unprecedented agricultural and public health challenge. Critically, the mouth-to-teat transmission mechanism reveals that traditional isolation of infected individuals is insufficient. All cattle with nursing relationships must be isolated simultaneously. Importantly, separating suckling calves from lactating cows during outbreaks represents a vital intervention to limit intra-herd spread. Combined with targeted vaccination, these approaches provide practical solutions for effective H5N1 control and reduced zoonotic risk.

## References

[1] Shi J, Kong H, Cui P, et al. H5n1 virus invades the mammary glands of dairy cattle through “mouth-to-teat” transmission. Natl Sci Rev. 2025; Jul 1;12(9):nwaf262. doi:10.1093/nsr/nwaf262 PMC12342610 40809875

[2] Keil NM, Audigé L, Langhans W. Is intersucking in dairy cows the continuation of a habit developed in early life?. J Dairy Sci. 2001;84(1):140–146. doi:10.3168/jds.S0022-0302(01)74462-1 11210026

[3] Lidfors L, Isberg L. Intersucking in dairy cattle—review and questionnaire. Appl Anim Behav Sci. 2003;80(3):207–231. doi:10.1016/S0168-1591(02)00215-0

[4] Lakdawala SS, Jayaraman A, Halpin RA, et al. The soft palate is an important site of adaptation for transmissible influenza viruses. Nature. 2015;526(7571):122–125. doi:10.1038/nature15379 26416728 PMC4592815

[5] Hatta M, Gao P, Halfmann P, et al. Molecular basis for high virulence of Hong Kong H5N1 influenza A viruses. Science. 2001;293(5536):1840–1842. doi:10.1126/science.1062882 11546875

[6] Liang L, Jiang L, Li J, et al. Low polymerase activity attributed to PA drives the acquisition of the PB2 E627K mutation of H7N9 avian influenza virus in mammals. mBio. 2019 Jun 18;10(3):e01162–19. doi:10.1128/mBio.01162-19. PMC6581862 31213560

[7] Kong H, Ma S, Wang J, et al. Identification of key amino acids in the PB2 and M1 proteins of H7N9 influenza virus that affect Its transmission in Guinea pigs. J Virol. 2019;94(1):10–1128. doi:10.1128/jvi.01180-19 PMC6912098 31597771

[8] Shi J, Deng G, Kong H, et al. H7n9 virulent mutants detected in chickens in China pose an increased threat to humans. Cell Res. 2017;27(12):1409–1421. doi:10.1038/cr.2017.129 29151586 PMC5717404

[9] Shi J, Zeng X, Cui P, et al. Alarming situation of emerging H5 and H7 avian influenza and effective control strategies. Emerg Microbes Infect. 2023;12(1):2155072. doi:10.1080/22221751.2022.2155072 36458831 PMC9754034

[10] Zeng X, Tian G, Shi J, et al. Vaccination of poultry successfully eliminated human infection with H7N9 virus in China. Sci China Life Sci. 2018;61(12):1465–1473. doi:10.1007/s11427-018-9420-1 30414008

[11] Maines TR, Chen L-M, Van Hoeven N, et al. Effect of receptor binding domain mutations on receptor binding and transmissibility of avian influenza H5N1 viruses. Virology. 2011;413(1):139–147. doi:10.1016/j.virol.2011.02.015 21397290 PMC5470842

